# Presence of SARS-CoV-2 and Its Entry Factors in Oral Tissues and Cells: A Systematic Review

**DOI:** 10.3390/medicina57060523

**Published:** 2021-05-23

**Authors:** Marco Felipe Salas Orozco, Nereyda Niño-Martínez, Gabriel-Alejandro Martínez-Castañón, Nuria Patiño Marín, Carolina Sámano Valencia, Farid Alonso Dipp Velázquez, Paulina del Carmen Sosa Munguía, Miguel Angel Casillas Santana

**Affiliations:** 1 Doctorado en Ciencias Odontológicas, Facultad de Estomatología, Universidad Autónoma de San Luis Potosí, San Luis Potosí C.P. 78290, Mexico; nuriapaty@uaslp.mx; 2 Facultad de Ciencias, Universidad Autónoma de San Luis Potosí, San Luis Potosí C.P. 78210, Mexico; nereyda.nino@uaslp.mx (N.N.-M.); mtzcastanon@fciencias.uaslp.mx (G.-A.M.-C.); 3 Maestría en Estomatología con Opción Terminal en Ortodoncia, Facultad de Estomatología, Benemérita Universidad Autónoma de Puebla, Puebla C.P. 72410, Mexico; carolina.samano@correo.buap.mx (C.S.V.); farid.dipp@correo.buap.mx (F.A.D.V.); 4 Residente de la Maestría en Ciencias Médicas e Investigación, Facultad de Medicina, Benemérita Universidad Autónoma de Puebla, Puebla C.P. 72410, Mexico; paulina.sosam@alumno.buap.mx

**Keywords:** SARS-CoV-2, oral tissues, entry factors, TMPRSS, furin, ACE2, COVID-19

## Abstract

*Background and Objectives*: The aim of this systematic review is to summarize the current data about the presence of severe acute respiratory syndrome coronavirus 2 (SARS-CoV-2) and its entry factors in oral tissues and cells. *Materials and Methods*: This systematic review was carried out based on the Preferred Reporting Items for a Systematic Review and Meta-Analysis (PRISMA). Three databases were analyzed (Pubmed, Web of science and Scopus) by three independent researchers. From the 18 identified studies, 10 of them met the inclusion criteria. The presence of SARS-CoV-2 or its entry factors (angiotensin-converting enzyme II (ACE2), transmembrane serine proteases (TMPRSS), and furin) was analyzed in these 10 studies during the pandemic. *Results:* ACE2 expression was analyzed in 9 of the 10 studies. ACE2 is expressed mainly in the tongue, oral mucosa, salivary glands and epithelial cells. The expression of the TMPRSS2 gene or protein was analyzed in 6 studies. These studies reported that the expression of TMPRSS2 was mainly in the salivary glands, tongue, sulcular epithelium and oral mucosa; as well as in cells of the salivary glands (ductal, acinar and myoepithelial cells) and the tongue (the spinous-based cell layer, horny layer and the epithelial surface). Other TMPRSS were also reported. The expression of TMPRSS3, TMPRSS4, TMPRSS5, TMPRSS7 and TMPRSS11D was reported mainly in salivary glands and in epithelial-type cells. Furan expression was analyzed in three studies. The expression of furin was detected mainly in epithelial cells of the tongue. A variety of methods were used to carry out the detection of SARS-CoV-2 or its input molecules. *Conclusions*: These results show that SARS-CoV-2 can infect a wide variety of oral tissues and cells, and that together with the theories dedicated to explaining the oral symptoms present in SARS-CoV-2 positive patients, it provides us with a good scientific basis for understanding the virus infection in the oral cavity and its consequences.

## 1. Introduction

Wuhan, China, was the first city in which the strain of the new coronavirus (severe acute respiratory syndrome coronavirus 2 (SARS-CoV-2)) was identified, and the virus has caused a global pandemic since December 2019. The respiratory disease caused by SARS-CoV-2 was named as coronavirus disease 19 (COVID-19) by the World Health Organization (WHO) [1]. By 10 March 2021 there had been 117,332,262 confirmed cases of COVID-19, including 2,605,356 deaths, reported to the WHO. As of 9 March 2021 a total of 268,205,245 vaccine doses had been administered [2]. The pandemic has had a strong impact on the health systems and economies of most of the countries of the world. One of the main criteria for the diagnosis of SARS-CoV-2 is its detection by polymerase chain reaction (PCR) from oropharyngeal swabs [3]. However, the presence of SARS-CoV-2 has been reported in a wide variety of human tissues, cells, secretions and excretions (like sputum, faeces and urine) [4]. The most common symptoms of COVID-19 are fever, dry cough, headache, myalgia, fatigue and loss of taste and smell. Likewise, the virus has the ability to cause extensive lung damage and cause death in patients with comorbidities or poor health [5]. SARS-CoV-2 was initially isolated from human airway epithelial cells [6]. Later, it was discovered that the SARS-CoV-2 genome is very similar to the genomes of the bat-SL-CoVZC45 and bat-SLCoVZXC21 viruses; however, the external subdomain of the SARS-CoV-2 receptor-binding domain (RBD) was more similar to that of the SARS-CoV [7]. Due to this, it was hypothesized that the entry route of SARS-CoV-2 to cells was through the cellular receptor for angiotensin-converting enzyme II (ACE2), since this is the one used by viruses such as SARS-CoV and HCoV-NL63. This was confirmed by later studies that reported that the SARS-CoV-2 spike protein has a high affinity for the ACE2 cellular receptor [8]. Therefore, cells that express the ACE2 receptor are susceptible to being infected by SARS-CoV-2 [9]. Furin is a protein convertase synthesized by host cells, and this protease can be used by SARS-CoV-2 to regulate its mechanism of entry into cells. Furin cleaves the SARS-CoV-2 Spike (S) protein at two specific sites: S1 and S2. This separates the two major domains of protein S during the entry of the virus into host cells. The S1 domain has a function related to receptor binding, while the S2 domain regulates the fusion of the virus with the host cell [10]. Another type of proteases that participate in the mechanism of entry of SARS-CoV-2 into host cells are the transmembrane serine proteases (TMPRSS). These belong to a family of 17 members, which is subdivided into four subfamilies (HAT/DESC, epsin/TMPRSS, matriptase and Corin). Most of the TMPRSS that have been reported to participate in the SARS-CoV-2 entry mechanism belong to the epsin/TMPRSS subfamily [11].

The aim of this systematic review is to summarize the current data about the presence of SARS-CoV-2 and its entry factors in oral tissues and cells.

## 2. Materials and Methods

This systematic review was carried out based on the Preferred Reporting Items for a Systematic Review and Meta-Analysis (PRISMA). Three databases were analyzed, Pubmed, Web of science and Scopus. The search was carried out from September 2019 to February 2021. The search terms used were: SARS-CoV-2 and Oral cells, SARS-CoV-2 and Oral cavity, SARS-CoV-2 and Oral cells, SARS-CoV-2 and Oral Tissues, SARS-CoV-2 and dental cells. Studies were selected by three independent researchers by first reviewing the title and abstract. After this first review, the studies that were deemed to meet the inclusion criteria were selected for further analysis.

Both studies published in English in peer review journals, letters to the editor and preprints were included due to the constant generation of new information during the development of the pandemic. The selected studies had to include human studies, determining the presence or expression of SARS-CoV-2 or its entry factors in oral tissues. The articles also had to include the techniques for analyzing the presence or expression of SARS-CoV-2 and its entry molecules. The exclusion criteria were articles that were not in the English language, studies that carried out experiments on animals, and articles that were not accessible.

The presence of bias in the studies included in this systematic review was evaluated by the modification of the Cochrane tool reported by Koletsi et al. [12]. One reviewer (M.A.C.S.) assessed basic elements to identify potential bias, which was then duplicated by a second reviewer (M.F.S.O). Any inconsistencies were straightened out with the help of a third reviewer (P.C.S.M.).

The quality of the “in vivo” studies included in this systematic review was assessed using the guidelines reported by Hadley et al. [13].

## 3. Results

**Figure 1:**
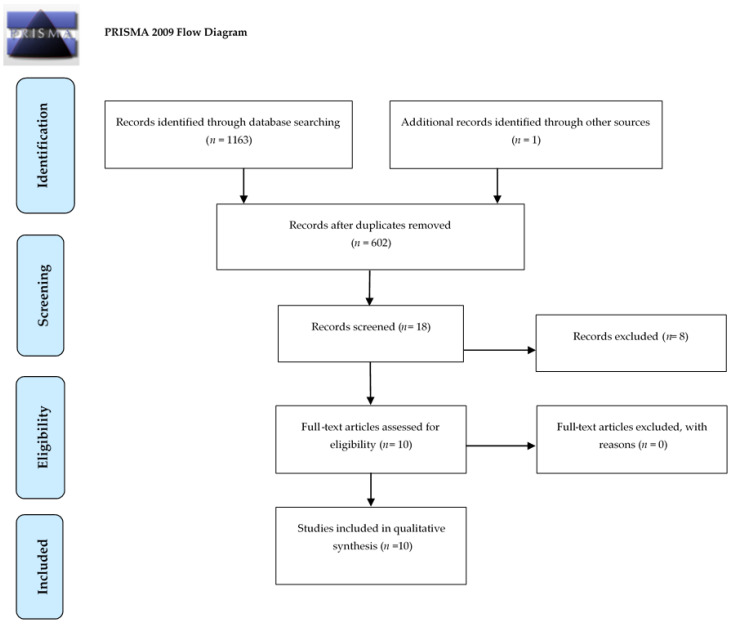
The Preferred Reporting Items for a Systematic Review and Meta-Analysis (PRISMA) flow diagram of record processing and elimination.

Selection process is summarized by the flow-chart reported in Figure 1. The results of the search in the three different databases provided 18 studies. From these 18 studies, 10 of them met the inclusion criteria. Nine of the 10 studies focused on analyzing the expression of the entry molecules of SARS-CoV-2 in oral cells and tissues. The entry molecules studied in these 9 studies were: ACE2, TMPRSS2, TMPRSS3, TMPRSS4, TMPRSS5, TMPRSS7, TMPRSS11D and furin. In only one of the 10 studies were both human and mouse samples analyzed. Only one study focused on identifying the presence of SARS-CoV-2 in oral tissues by means of PCR. The study design most used was “in silico”, followed by “in vivo” and finally “in vitro” [14,15,16,17,18,19,20]. Most of the articles showed a combination of two or more of the aforementioned study designs.

### 3.1. Angiotensin-Converting Enzyme II (ACE2) Expression

ACE2 expression was analyzed in 9 of the 10 studies mentioned above. Xu et al. reported that ACE2 expression was significantly higher in the oral tongue compared to other oral tissues (floor of mouth, base of tongue and other sites). These authors also reported the expression of ACE2 in oral cells (fibroblast epithelial cells) and cells of the immune system (B and T cells). In general, the highest expression of ACE2 is found in epithelial cells of the oral tongue [21].

Zhong et al. reported that of the total oral cells, cells capable of expressing the ACE2 receptor represented 2.2%. Of this 2.2%, 92% were epithelial cells. The results of the immunohistochemistry showed that the anatomical areas with the highest expression of ACE2 in the oral mucosa, from highest to lowest were: lip, tongue, buccal mucosa, gingival and palatal tissue. The cells of these anatomical locations that most expressed ACE2 were the epithelial cells of the basal layer, followed by the fibroblasts and the endothelial cells [20]. Song et al. reported a moderate expression of the ACE2 gene in the salivary glands. In this study, no significant difference in ACE2 expression was reported in different age ranges or between women and men [19].

Sakaguchi et al. reported a different ACE2 expression between the layers of squamous epithelium of the tongue evaluated through immunohistochemistry. ACE2 was also reported in the epithelial cells of the taste buds. The expression of ACE2 was also reported in the squamous gingival epithelium, mainly in the cytoplasm and nucleus of the spinous basal layer. In the submandibular gland the expression of ACE2 was observed in the ductal epithelium and in the serous cells. The same authors detected the expression of ACE2 through real-time polymerase chain reaction (RT-PCR) in cell cultures of fungiform taste buds [17].

Huang et al. reported the expression of ACE2 in 8 cell types of the mucosal epithelium (basal 1, basal 2, basal cycling, suprabasal, serous acini, mucous acini and myoepithelium) [22]. Pascolo et al. reported the expression of the ACE2 gene in salivary glands, however, the ACE2 protein cannot be detected in this oral tissue [16]. Galicia et al. reported the expression of ACE2 in pulp tissue, and found that said expression is not affected by inflammatory phenomena [14]. Sawa reported the expression of ACE2 in tongue, lip and cheek [18]. Finally, Chen et al. reported the expression of ACE2 in salivary glands [23].

### 3.2. Transmembrane Serine Proteases (TMPRSS) Expression

#### 3.2.1. TMPRSS2 Expression

The expression of the TMPRSS2 gene or protein was analyzed in 6 studies. Song et al. reported that the expression of TMPRSS2 is high in the salivary glands (parotid and submandibular) and oral mucosa. This expression was different between women and men. Likewise, the main cells of the salivary glands that expressed TMPRSS2 were base, ductal, acinar and myoepithelial cells [19]. Sakaguchi et al. through immunohistochemistry reported the presence of TMPRSS2 in the epithelial cells of the taste buds, spinous and degenerate cells of the squamous epithelium of the tongue and in 3 layers (the spinous-based cell layer, horny layer and the epithelial surface) of the gingival squamous epithelium. TMPRSS2 was also detected in the sulcular epithelium. Finally, TMPRSS2 was detected in the ductal and serous cells of the submandibular glands. The results of the Western-blot indicated the presence of TMPRSS in samples of saliva and tongue coating. Finally, the RT-PCR reported the expression of TMPRSS2 in fungiform papilla cells [17]. Huang et al. reported the co-expression of TMPRSS2 with ACE2 in 8 cell types (from highest to lowest expression: basal 1, ductal cells, myoepithelial cells, mucous cells, serous cells, cycling and basal 2) derived from salivary glands and oral mucosa [22]. The expression of TMPRSS2 was also reported in pulp tissue [14]. Sawa et al. through RT-PCR reported a high expression of TMPRSS2 in the lingual and buccal mucosa. The Immunostaining analysis reported the expression of TMPRSS2 in the keratinized and non-keratinized stratified squamous epithelium, in the spinous and granular stratum, and in the serous and mucosal acini [18].

#### 3.2.2. Other TMPRSS

Although TMPRSS2 is the one that has been most related to ACE2 and the mechanism of virus entry into cells, two articles reviewed the presence of other types of TMPRSS. Song et al. reported that TMPRSS3, TMPRSS5 and TMPRSS7 correlate with the expression of ACE2 in salivary glands. Huang et al. also reported the expression of TMPRSS4 and TMPRSS11D in the 8 types of epithelial cell that they identified [22]. These authors also reported the expression of endosomal proteases (CTSB, CTSL and BSG) in 33 cell clusters belonging to epithelial tissue, lamina propia and cells of the immune system [19].

### 3.3. Furin Expression

Furin expression was analyzed in three studies. Zhong et al. reported that Furin mRNA was expressed in the following oral cells from highest to lowest: epithelial cells, fibroblasts, T cells, and endothelial cells. Likewise, through immunostaining analysis, the furin protein was detected mainly in the lip, tongue and gingiva, followed by the palatal and buccal mucosa. Of these tissues, the spiny layer was the one with the highest presence of furin [20]. Through immunohistochemical analysis, Sakaguchi et al. reported the presence of furin in the spinous and basal layer of the squamous epithelium of the tongue. Furin was also detected in the lower layers of the taste buds. In the squamous gingival epithelium, furin was detected mainly in the basal cells and on the buccal surface of the sulcular epithelium [17]. On the other hand, the Western-blot analysis revealed the presence of furin in saliva. Likewise, the expression of furin was detected in fungiform taste buds by means of RT-PCR [17]. Huang et al. reported the expression of furin in 33 clusters of cells grouped into three groups (mucous epithelium, lamina propia and cells of the immune system) [22].

**Figure 2:**
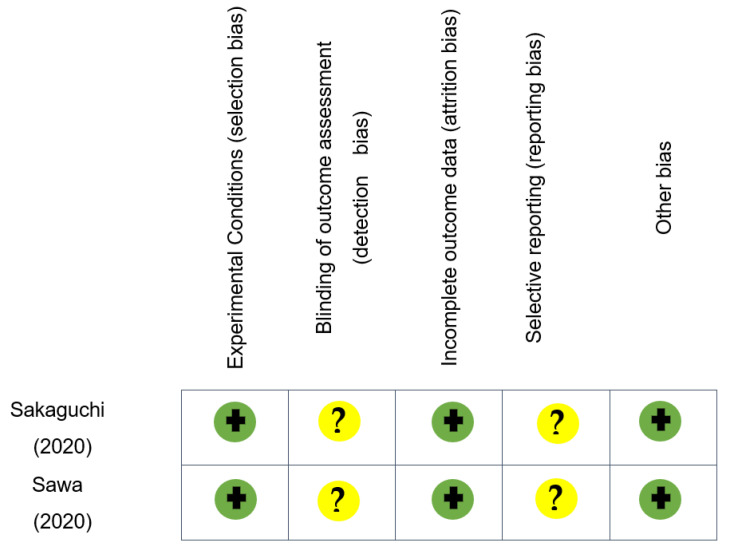
Risk of bias for each of the included in vitro studies (*n* = 2). The green circles with plus signs indicate a low risk of bias; the yellow circles with a question mark indicates an unclear risk of bias in the studies.

The main results of the studies included in this systematic review are shown in Table 1. The results of the evaluation of the presence of bias and the quality of the studies are shown in Figure 2 and Table 2, respectively. There is no instrument to assess the risk of bias or the quality of the “in silico” studies.

## 4. Discussion

The clinical manifestation of SARS-CoV-2 can range from an asymptomatic course to severe lung damage and death. The factors involved in the development of the different clinical presentations of the SARS-CoV-2 infection are still unknown. To infect tissues and organs, SARS-CoV-2 has mechanisms to enter the different cell types that compose them. The entry of SARS-CoV-2 into cells occurs mainly through the ACE2 and TMPRSS receptors [24]. Understanding the viral infection pathways and the tissues vulnerable to COVID-19 infection is crucial to comprehending the infection’s clinical symptoms. Although studies of SARS-CoV-2 infection in oral tissues are scarce, this systematic review clarifies some points regarding infection in oral tissues and the clinical symptoms that may occur.

According to the results of this systematic review, ACE2 and TMPRSS receptors are expressed in a great variety of oral tissues and cells, mainly in the epithelial cells of the tongue and major and minor salivary glands. Oral cells and tissues sensitive to infection by SARS-CoV-2 coincide with the anatomical sites in which clinical oral symptoms have been reported in patients positive to infection by SARS-CoV-2. For example, Chen et al. reported that the most common oral symptoms in 108 patients positive for SARS-CoV-2 infection were dry mouth (46.3%) and amblygeustia (47.2%) [23]. Brandao et al. published a series of cases in which 8 patients positive for SARS-CoV-2 infection first presented dysgeusia and later developed oral ulcers (mainly necrotic ulcers and aphthous ulcers) in the oropharynx, palate, lips and tongue [25]. Tapia et al. reported 4 cases of patients with SARS-CoV-2 infection in which the oral symptoms they presented were non-specific mucositis and bullous hemorrhagic angina-like lesion on the tongue and palate [26]. Hjelmesæth et al. reported 3 members of a family with SARS-CoV-2 infection who had taste alterations [27]. Some authors report that SARS-CoV-2 infection can contribute to the appearance of a disease very similar to Kawasaki disease in children, which includes some oral symptoms. It is thought that this is due to the storm of pro-inflammatory cytokines caused by the virus infection [28,29].

So far, in the literature there is some theories aimed at explaining the oral symptoms caused by the SARS-CoV-2 infection, and which seem to have some support with the results presented in this systematic review. Finsterer and Stollberger in a letter to the editor proposed four theories. The first two involve the central nervous system (CNS) and the peripheral nervous system (PNS), respectively [30]. Viruses can invade the human nervous system through three mechanisms: peripheral nerve infection, CNS infection using axonal transport as a route of entry, or infecting red blood cells which help the virus bypass the blood-brain barrier [31]. The first theory involves the CNS since SARS-CoV-2 has shown the potential to invade the CNS, especially in severe cases. In addition, in this type of patient, the presence of the virus has been reported even in the cerebrospinal fluid. The authors propose that the virus could cause local meningitis that compromises the gustatory-cortex. In the same way, the PNS is sensitive to infection by SARS-CoV-2, specifically in most of the cranial nerves (I, II, III, IV, VI, VII, IX and X) [30]. It has been reported that one of the main cranial nerves that contributes to infection and spread of viruses to the nervous system is the vagus nerve [32].

In the second hypothesis, the authors propose that the invasion by the virus of oral cells, mainly the epithelial cells of the tongue and taste buds, can cause a local stomatitis that would lead to an alteration of the function of the taste buds [30]. This theory seems to be supported to some extent by the results of this systematic study. In the third hypothesis, the authors propose that the presence of the virus triggers an immune reaction that leads to the production of antibodies that can attack not only the virus but also the cells of the oral cavity, which would again include cells of the taste buds. This theory seems to be supported by “in silico” studies in which it was determined that type 2 taste receptors participate in the immune response [33]. In the fourth theory, the authors suggest that alterations in taste and smell in patients infected by SARS-CoV-2 may be due to the drugs used to combat the said infection. This is because some of the drugs show these types of alterations as side effects [34]. Finally, in the last theory the authors propose that the virus has the ability to directly infect the cells present in the taste buds causing the alteration of the sense of taste in patients infected with SARS-CoV-2 [30].

Vaira, Salzano, Fois, et al. in a letter to the editor, propose two hypotheses that could help explain or relate the infection of the cells of the cavity by SARS-CoV-2 and the appearance of oral symptoms during the said infection [35]. In the first hypothesis, the authors mention that it has been recently shown that SARS-CoV-2 has the ability to bind to sialic acid receptors [36]. This would cause a decrease in the amount of sialic acid, which is a component of salivary mucin. Salivary mucin has the function of protecting the glycoproteins in charge of carrying the molecules that provide flavor to the taste pores. Therefore, a decrease in sialic acid would cause an alteration of the saliva mucin and its functions, which would cause the accelerated degradation of the flavor molecules, which would result in the appearance of ageusia [35]. The second theory mentions that ageusia could be due to the infection present in the cells of the respiratory tract and the intimate relationship of these two sensory systems, however, this seems unlikely because the prevalence of ageusia (22.5%) [37] and anosmia (10.2%) [38] are different.

Nataf proposes a hypothesis in which, through an “in silico” study, it was determined that there is a co-regulation of ACE2 and dopa decarboxylase (DDC). DDC is an important enzyme that participates in the synthesis of neurotransmitters like histamine, dopamine and serotonin. Therefore, an alteration in the expression of ACE2 by SARS-CoV-2 could cause an alteration in the expression of DDC, and likewise an alteration in the levels of the neurotransmitters; and the appearance of symptoms such as ageusia [39]. On the other hand, Mariz et al. propose a hypothesis in which SARS-CoV-2, by binding to ACE2 receptors, causes a decrease in the expression of ACE2 (which has as one of its functions, the degradation of angiotensin II [Ang-II]). Therefore, Ang-II would not degrade and accumulate in the taste buds causing the appearance of dysgeusia [40]. Galvan Casas et al. propose a theory to explain the appearance of ulcers in the oral mucosa. According to the authors, the ulcers would be caused due to a co-infection of bacteria, fungi and viruses [41]. This theory seems to be supported by recent studies. For example, Lin et al. reported viral coinfection of SARS-CoV-2 positive patients [42]. Another study in China reported that 5.8% of SARS-CoV-2 positive patients had co-infection with other coronaviruses and influenza viruses [43]. Likewise, a study in Italy reported that 11% of SARS-CoV-2 positive patients had bacterial and fungal co-infections [44].

Sarode et al. propose a hypothesis that aims to explain gustative symptoms and mouth ulcers in patients positive for SARS-CoV-2. In their hypothesis, the authors propose that red blood cells express receptors (ACE2, CD147, and CD26) for the entry of SARS-CoV-2, which would cause hemolysis. Likewise, SARS-CoV-2 mimics hepcidin, which increases ferritin levels causing iron deficiency and anemia [45]. It is likely that in these mechanisms the deleterious effects of SARS-CoV-2 on microcirculation are also involved [46]. Sawa et al. reported the expression of ACE2 in cells of the lingual and buccal mucosa by means of RT-PCR. Likewise, through immunostaining, they also reported the presence of ACE2 in the keratinized and non-keratinized stratified squamous epithelium. In the labial glands, the expression of ACE2 was detected in the serous and mucous acini [18]. Finally, Amirfakhryan proposes a hypothesis to explain the appearance of a disease very similar to Kawasaki disease in children. In this, the author proposes that children with a genetic predisposition to develop Kawasaki disease (because they have a genetic under-expression of ACE2), develop the disease since SARS-CoV-2 increases this under-expression by joining the ACE2 receptors during their cellular infection mechanism [28].

Two articles included in the results of this systematic review report the presence of SARS-CoV-2 in periodontal tissues. This seems to coincide with the report of SARS-CoV-2 in gingival crevicular fluid [47]. However, the reports of SARS-CoV-2 from periodontal cells and tissues are lower compared to the tongue and salivary glands, so this seems to contradict the theory that periodontal health can significantly influence the development of the SARS-CoV-2 infection [48]. For example, Roganović reports that it is possible that the presence of diabetes and periodontitis in patients infected by SARS-CoV-2 causes an increase of two microRNAs (-146a and -155) which in turn causes a greater expression of the ACE2 receptor. This seems to favor the mechanisms of SARS-CoV-2 infections in periodontal tissues; however, this study was carried out through a bioinformatics analysis, so clinical or in vitro studies are needed to confirm these findings [49]. Marouf et al. determined that the presence of periodontitis in SARS-CoV-2 positive patients is associated with a greater severity of the disease and an increased risk of death [50].

Likewise, some of the articles included in the results of this systematic review report the presence of SARS-CoV-2 or its entry factor ACE2 in saliva; this is due to the presence of degenerated cells present in it. This helps to explain the presence of the virus in saliva and the use of saliva as a sample for virus detection in diagnostic tests [51]. Most of the selected articles use the bioinformatic analysis to obtain their results, and this is due to the danger that the realization of clinical trials represents. Studies “in silico” offer a great advantage, since through the analysis of a large amount of information, (most of it, previously generated) significant results can be obtained that serve to focus clinical studies more effectively. However, the results of “in silico” studies still have to be corroborated by clinical studies [52]. One of the studies included in the results of this article has a preprint format, and although this format has the advantage of making the information accessible more quickly, one of its disadvantages is that these papers have not been peer-reviewed, and therefore, they may contain information that is not validated [53].

## 5. Conclusions

The results of this systematic review help us to better understand the mechanisms of SARS-CoV-2 infection in oral tissues and cells. Likewise, by integrating this information with the theories that explain the symptoms related to the infection of SARS-CoV-2 in the oral cavity, it is possible to better understand the pathophysiology and symptoms of this virus at the stomatological level.

## Figures and Tables

**Table 1:** Studies that report the presence of the molecules responsible for the severe acute respiratory syndrome coronavirus 2 (SARS-CoV-2) infection in oral tissues and cells.

First Author	Year	Design	Samples	Methods	Molecules	Main Finding
Xu	2020	In silico	Humans	RNA sequencing (RNA-seq)	Angiotensin Converting Enzyme 2 (ACE2)	High expression of ACE2 receptor in tongue
Song	2020	In silico	Humans	RNA-seq	ACE2 Transmembrane protease serine 2 (TMPRSS2)	ACE2 and TMPRSS2 are expressed in salivary glands
Sakaguchi	2020	In silico	Humans	Real-Time Polymerase Chain Reaction(RT-PCR)	ACE2	ACE2, TMPRSS2 and furin are expressed in tongue,
		In vitro		Western Blot	Furin	gingiva, taste buds and saliva
		In vivo		Immunohistochemistry	TMPRSS2	
Huang	2020	In silico	Humans	RNA-seq	ACE2	SARS-COV-2 entry factors are expressed in salivary
		In vivo		In situ hybridization	TMPRSS2	glands, mucosa and saliva
					TMPRSS4	
					TMPRSS11D	
Fernandes Matuck	2021	In vivo	Humans	RT-PCR	E and	SARS-CoV-2 was detected in periodontal tissue
				Histopathological analysis	RdRp genes	(junctional epithelium, adjacent oral gingival
						epithelium and connective tissue)
Pascolo	2020	In silico	Humans	RNA-seq	ACE2	ACE2 and TMPRSS RNA and proteins were found
				Protein Expression	TMPRSS2	in salivary glands
Galicia	2020	In silico	Humans	RNA-seq	ACE2	ACE2 and TPMRSS2 are expressed in the dental pulp
					TMPRSS2	
Sawa	2020	In vitro	Humans	RT-PCR	ACE2	ACE2 and TMPRSS2 expression was detected in
		In vivo	Mice	Microarray	TMPRSS2	tongue, lip and cheek
				Immunostaining		
Zhong	2020	In silico	Humans	RNA-seq	ACE2	ACE2 and Furin expression on epithelial cells from
				Immunohistochemistry	Furin	different oral anatomical sites
Chen	2020	In silico	Humans	RNA-seq	ACE2	ACE2 expression in salivary glands
		In vivo		RT-PCR		

**Table 2:** Quality assessment of the reports included in this systematic review.

Item	Sakaguchi	Huang	Fernandes Matuck	Sawa	Chen
	2020	2020	2021	2020	2020
1	2	2	2	2	2
2	2	2	1	2	2
3	2	2	1	2	0
4	2	2	1	2	0
5	2	2	2	2	2
6	2	2	1	1	2
7	2	2	0	0	0
8	2	2	0	2	0
9	2	2	1	2	1
10	2	2	1	2	2
Score	20	20	10	17	11

Quality Assessment (1) Question/objective sufficiently described? (2) Design appropriate to answer the study question? (3) Samples, reagents, assays sufficiently described? (4) Methods described in sufficient detail? (5) Study can be easily replicated? (6) Sample/experiment number sufficient for internal validity? (7) Appropriate negative controls? (8) Appropriate statistical analysis? (9) Results reported in sufficient detail? (10) Do the results support the conclusion? Scoring: Fully compliant = 2 points, Partially compliant = 1 point, No = 0; Total score: 17–20 = High quality, 11–16 = Regular quality, 0–10 = Low quality.

## Data Availability

Not applicable.
